# Open-label randomized controlled trial to compare wound dressings for patients undergoing hip and knee arthroplasty: study protocol for a randomized controlled trial

**DOI:** 10.1186/s13063-018-2755-8

**Published:** 2018-07-05

**Authors:** Maria López-Parra, Dolors Gil-Rey, Esmeralda López-González, Eva-Maria González-Rodríguez, Isabel Simó-Sánchez, Francisco Zamora-Carmona, Lidia Roqueta-Andreu, Marta Arizu-Puigvert, Dolors Abril-Sabater, Àngels Moreno-Álvarez, Àngels López-Bonet, Gisela López-Hidalgo, Helena Costa-Ventura, Laura García-Pardo, Mireia Rico-Liberato, Miriam García-Borràs, Maria Teresa Arnal-Leris, Mònica Sianes-Gallén, Roser Vives

**Affiliations:** 1 Parc Taulí Hospital Universitari, Institut d’Investigació i Innovació Parc Taulí I3PT, Universitat Autònoma de Barcelona, Parc Taulí 1, 08208 Sabadell, Barcelona, Spain; 2 Capresa, prevención de riesgos laborales, C/ Badajoz, 145, 08018 Barcelona, Spain; 3 Departament de Farmacologia, de Terapèutica i de Toxicologia, Universitat Autònoma de Barcelona, Cerdanyola del Vallés (Barcelona), Spain

**Keywords:** Occlusive dressings, Nursing, Arthroplasty, Replacement, Knee, Hip (MeSH)

## Abstract

**Background:**

Surgical wounds are covered to prevent bleeding, absorb the exudates, and provide a barrier against external contamination. Currently, in our hospital, after orthopedic surgery, traditional occlusive dressing of sterile gauze and non-woven hypoallergenic adhesive tape is placed. Some of the newest dressings have been shown to reduce the incidence of blisters compared with traditional dressing or colloid adhesive dressings. However, there are no comparative evaluations between the different types of dressings and their contribution to the overall results of the healing process.

**Methods/design:**

This is a randomized, controlled, open-label trial to compare five types of dressings used in total knee and hip arthroplasty surgical wounds. A total of 550 patients will be randomly allocated to one of the following dressings: (1) traditional occlusive dressing, (2) Aquacel Surgical^®^, (3) Mepilex^®^ Border Post-Op, (4) OpSite Post-Op Visible, or (5) UrgoTul^®^ Absorb Border. The dressing assigned is placed right after surgery. Patients will be followed up to 14 days after surgery when the dressing is definitively removed and will be tracked up to 3 months to record any late complications. During the immediate postoperative period and patient hospitalization and at the ambulatory visits after discharge, every time that the dressing is changed, nurses perform the study assessments. The main study outcome will be the percentage of patients with skin integrity at all times when the dressing has been changed. Skin integrity is a composite of the absence of blisters, erosion, erythema, maceration, swelling, wound dehiscence, and purulent exudates. Secondary outcomes include time to first change of dressing; percentage of patients with presence/absence of blisters, erosion, erythema, maceration, swelling, wound dehiscence, and purulent exudates; number of dressing changes needed; days of hospital stay; and nurse and patient satisfaction.

Differences in the main variable between each treatment group and group 1 will be tested by means of a chi-squared test or Fisher’s exact test. Subgroup analyses of diabetic and non-diabetic patients, patients with a body mass index of more than 30 or not more than 30, and type of surgery (hip or knee) are planned.

**Discussion:**

The results of this study will be useful for clinical decision making by giving information on the contribution of the dressings studied to the outcome of the wound and may also show which dressing offers better results depending on the characteristics of patients.

**Trial registration:**

This trial has been registered at ClinicalTrials.gov (NCT03190447). Retrospectively registered on 16 June 2017.

**Electronic supplementary material:**

The online version of this article (10.1186/s13063-018-2755-8) contains supplementary material, which is available to authorized users.

## Background

Surgical wounds are covered to prevent bleeding, absorb the exudates, and provide a barrier against external contamination. It is recognized that not covering the surgical wound in some surgeries may increase the risk of complications [1]. Leaving the wound uncovered is especially discouraged in those patients with suboptimal conditions that do not guarantee that the skin can constitute a protective barrier [2].

Surgical wound infection is the main nosocomial infection affecting postsurgical patients [3]. In our setting, the rates of superficial wound infection in 2015 were 0.51% for total knee arthroplasty (TKA) and 0% for hip arthroplasty.

Currently, in our hospital, after orthopedic surgery, traditional occlusive dressing of sterile gauze and non-woven hypoallergenic adhesive tape is placed. In some cases, we have observed the appearance of blistering, a situation that increases the risk of infection, pain, and overall costs of the procedure [4, 5]. Surgical wounds after knee and hip arthroplasty are about 15 to 19 cm long and closed with staples, and in our center no drainage is placed. After a patient is discharged from the hospital, the staples are removed when the patient is ambulatory, 10 to 15 days after the intervention. During this period, the wound is covered.

In recent years, new wound dressings have come into the market, at increased cost, and thus it is important to assess their contribution to patients before its use can become widespread [6].

Potential postoperative complications include changes in skin integrity, such as erythema, erosion, maceration, and blistering, the last of these being considered the most important. Blistering consists of the separation of dermis and epidermis, probably caused by edema and inflammation, which usually appear after the fifth or sixth day of surgery [7], leading to increased wound pain and risk of infection. These complications reduce mobilization and thus prolong the time to recover and increase hospital stay. It has been advocated that the right placement of the wound dressing is one of the most relevant factors to avoid blistering. Other predisposing factors described in the literature are obesity, venous insufficiency, diabetes mellitus, and old age [7, 8]. The incidence of blistering described for orthopedic surgery varies from 6% to 24% [7, 9].

Some of the newest pre-formed dressings have been shown to reduce the incidence of blisters in comparison with traditional dressing or colloid adhesive dressings [10]. In a recent publication, Sharma et al. [11] reviewed 12 randomized clinical studies. The authors conclude that, compared with the traditional passive dressing, new dressings could be better in terms of wound complications, although no differences in terms of surgical site infections were detected. They also emphasize that the studies comparing different new dressings are limited. This systematic review and meta-analyses also showed that there is a wide variety of products studied in studies with a small sample size and thus they are not powered to detect differences in endpoints with low frequency. Measures of satisfaction, comfort, and pain reported by patients are seldom included in the studies and neither is the nurse’s opinion or the costs. The authors suggest that well-designed studies that tackle the limitations of the existing ones are needed.

In most cases, nurses are the first ones to assess the surgical wound and decide the type of dressing placed [12]. With the objective of assessing whether newer dressings present advantages over traditional ones, a team of nurses has initiated this randomized, open-label, parallel study comparing four different types of new dressings with the traditional one used in our setting in total knee replacement and total hip replacement.

## Methods/design

### Design

This is a randomized, controlled, single-center, open-label trial to compare four types of pre-formed dressings with the traditional one used in TKA and total hip arthroplasty (THA) surgical wounds. The trial will take place in Parc Taulí Hospital Universitari in Sabadell (Barcelona, Spain). Patients undergoing TKA or THA following the fast track will be randomly allocated to receive one of the following wound dressings (description in accordance with the manufacturers’ file): (1) traditional occlusive dressing of sterile gauze and non-woven hypoallergenic adhesive tape (Fixomull^®^, BSN medical, Hamburg, Germany); (2) Aquacel Surgical^®^ (ConvaTec, Deeside, UK), which has hydrocolloid technology that allows flexing with the skin as the body moves; its hydrofiber absorbs and locks in fluid and bacteria, and its polyurethane film provides a waterproof viral and bacterial barrier; (3) Mepilex^®^ Border Post-Op (Mölnlyke, Gothenburg, Sweden), which has absorbent fibers, very high flexibility, and polyurethane backing film and is shower-proof and provides a viral and bacterial barrier; it also has wide fixation borders; (4) OpSite Post-Op Visible (Smith & Nephew, London, UK), which has hydrocellular foam, allows monitoring of progress without unnecessary dressing changes, and has waterproof film that allows showering and transpiration and provides a barrier against bacteria; or (5) UrgoTul^®^ Absorb Border (Urgo Medical, Shepshed, UK), which has a silicone adhesive border that provides non-traumatic removal and is shower-proof; its absorbent polyurethane foam allows high fluid management without maceration.

### Study population

Patients older than 18 years undergoing primary TKA and THA in the fast track and with adequate cognitive ability will be informed by the nurse during the visit performed before surgery and will be invited to participate in the study. The fast track for elective orthopedic surgery consists of a coordinated initiative with the objectives of reducing the length of hospital stay and promoting faster recovery of patients. It consists of a pre-surgical preparation of the patient, in which comorbidities and surgical risk factors (i.e. anemia and hypoalbuminemia) are assessed and corrected. Patients following this track have been carefully selected and optimized before the surgical procedure. Procedures and assessments during hospital stay and postoperative follow-up are standardized to ensure fast recovery of patients. The process also gives guidance on surgical procedures, medication before and after surgery (i.e. antibiotic prophylaxis 30 min before surgery with one dose of cefuroxime 1500 mg for THA or cefonicid 2000 mg for TKA; in case of penicillin allergy, clindamycin 900 mg), and the anesthetic technique and intra- and post-operative analgesia. Patients who have damaged skin or who are not autonomous for activities of daily living will be excluded. Patients signing the informed consent form will be included in the study.

### Random assignment

Patients will be randomly assigned to one of the five groups. A computer-generated, permuted block, random list has been created by a designated member of our institution’s Clinical Trials Unit by using WinPepi etcetera module version 3.26. Sealed envelopes for individual patients have been created for each patient. The intervention will be assigned by a nurse by opening the sealed envelope during the visit before surgery, only after the patient has signed informed consent and after his or her eligibility has been fully checked. Age, gender, body mass index (BMI), and presence of diabetes will be recorded during this visit.

### Study procedures and assessments

On the day of surgery, the nurse in the operating room will place the dressing right after the surgery, as assigned. Information on the surgical procedure, such as length of surgery, total time of ischemia, use of a layer wound closure technique, and flexion or extension of the limb when applying the dressing (for TKA), will be recorded by operating theater nurses.

**Fig. 1:**
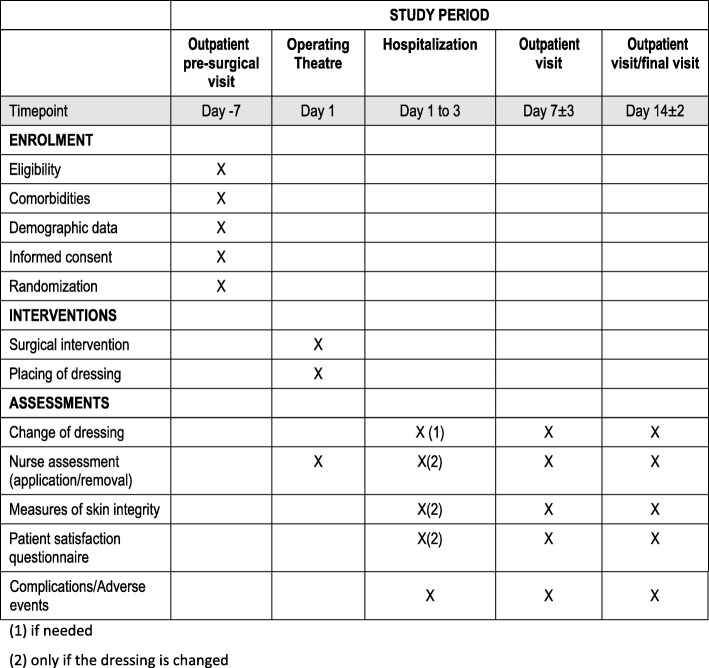
Schedule of enrolment, interventions, and assessments. (1) If needed. (2) Only if the dressing is changed

After that, patients will follow the fast track and will be discharged after 48 h in case of the absence of complications that require an extended stay. A outpatient visit is performed 7 days after the intervention. During this visit, the nurse changes the dressing and assesses the wound. On day 14 after surgery, a second nurse ambulatory visit is performed to remove the dressing, assess the wound, and remove the surgical staples. Assessment of wound during the ambulatory visits is performed by two different nurses from the study team in order to minimize the inter-observer variability. According to the current clinical practice in our hospital, during the immediate postoperative period and hospitalization, dressing is changed only in those cases when the surgeon requires it because of excessive bleeding or because of the presence of any other sign or symptom that is suggestive of wound complications. If this is the case, every time that the dressing is changed, nurses will perform the study assessments (Fig. 1. “Schedule of enrolment, interventions, and assessments”). The time spent in the procedure and the number of dressings used will also be recorded. In the event of serious health complications not related to the wound, patients will be withdrawn from the study. After the definitive removal of the dressing, patients will be tracked for the following 3 months to record any late complications, by review of the clinical records in order to detect any emergency ward visit, re-consultation, or re-hospitalization. Any untoward event detected during the follow-up (14 days) and a further 3 months will be followed up to resolution. In the event of wound complications, treatment needed (i.e. antibiotics and wound debridement) is recorded. Any other complications are recorded systematically and followed until resolution.

Direct costs of the dressings, the human resources needed for their use, and the economic cost of complications (treatments and emergency ward visits) will be assigned according to the prices in our institution and will be applied to the events of patients included in our study.

### Study outcomes

#### Primary outcome

The primary outcome is the percentage of patients in each treatment group with skin integrity at all times when the dressing has been changed. Skin integrity is a composite of the absence of blisters, erosion, erythema, maceration, swelling, wound dehiscence, and purulent exudates. All of these items are assessed by the nurse as presence or absence at the time of changing the dressing.

#### Secondary outcomes

Secondary outcomes are time to first change of dressing; percentage of patients with presence/absence of blisters, erosion, erythema, maceration, swelling, wound dehiscence, and purulent exudates; number of dressing changes needed during hospitalization; days of hospital stay; and total direct costs related to the surgical wound care.

Nurse and patient assessment questionnaires will also be assessed as secondary endpoints. The nurse’s questionnaire assesses ease of application and removal (0 to 5), and the patient questionnaire includes patient-reported pain at removal (11-point numerical rating scale) and limitations in being mobile and sitting, getting dressed and conducting personal hygiene, and resting and sleeping. Global satisfaction of nurses and patients will also be assessed. All of these assessments are 6-point rating scales (0–5) and are based on the ad-hoc questionnaires used by Springer et al. [13].

### Data collection and management

Study data will be collected in paper case report forms (CRFs) designed for the study. Risk-adjusted monitoring will be performed by personnel independent of the research team and will consist of checking all informed consent forms and completeness of all CRFs and source data verification for a random sample of patients. Data will be entered in the study database by the study team. Patients will be identified by a numerical code, and no personal information will be included in the paper CRF or the database.

### Statistical methods

#### Sample size

The estimation of sample size was based on the review of previous literature [11, 14]. Our hypothesis is that 82% of patients will present skin integrity with the traditional wound dressing (group 1) during the process of wound healing and that the application of any of the other dressings studied will result in 95% of patients with skin integrity. Given a two-sided risk of 5%, a power of 80%, and an expected loss of 15%, 110 patients per group are to be included. No adjustment for multiplicity has been considered, as the comparison of the different dressings with group 1 represents distinct research questions. All tests will be performed at an alpha level of 0.05.

#### Populations of analysis

The primary analysis will be performed according to the intention-to-treat principle and will include all randomly assigned subjects to whom the study dressing has been placed in the operation theater. Subjects will be considered and analyzed in the treatment group assigned. As no data imputation for missing values has been considered, only patients with available assessments of the main outcome will be considered in the main analysis. The final populations of analysis will be defined before the statistical analysis, and criteria will be applied to all patients regardless of the arm they have been assigned to.

#### Descriptive statistics

Patient characteristics will be described by treatment group and for the entire sample of patients. Quantitative variables will be presented as means and standard deviations or, in cases of skewed distribution, as medians and inter-quartile ranges. Categorical variables will be presented as absolute and relative frequencies.

#### Inferential statistics

Differences in the main variable between each treatment group and group 1 will be tested by means of a chi-squared test or Fisher’s exact test. For categorical ordinal variables, the analysis of variance (ANOVA) or Kruskal–Wallis test will be used. Time to event data will be summarized by means of Kaplan–Meier curves and compared with the log-rank test. Exploratory comparisons between the different new dressings will also be performed for primary and secondary variables. Subgroup analyses in diabetic and non-diabetic patients, patients with a BMI of more than 30 or not more than 30, and type of surgery (hip or knee) are planned.

### Feasibility

The number of TKAs and THAs following fast track was about 421 in 2016. Recruitment of patients started in April 2017 and is anticipated to end in April 2019. An investigator team composed mainly of nurses has been trained in all study procedures and is in charge of tracking and assessing the patient throughout the process, from ambulatory setting, operating room, and hospitalization.

### Ethics

This trial is being conducted in accordance with the Declaration of Helsinki and good clinical practice principles. The study protocol (version 1, December 2016) was approved by the research ethics committee of our center (*Comité Ético de Investigación Clínica de la Corporació Sanitària Parc Taulí*) in January 2017. The protocol has been registered at ClinicalTrials.gov (NCT03190447). Written informed consent will be obtained from each participant before any trial-related procedures are carried out. The present study protocol has been written in accordance with the Standard Protocol Items: Recommendations for Interventional Trials (SPIRIT) 2013 statement for reporting a clinical trial protocol [15]. The SPIRIT checklist is provided in Additional file 1.

## Discussion

We have designed a randomized clinical study to compare four new wound dressings with the traditional one in patients undergoing programmed TKA or THA in terms of skin integrity during the postoperative period. Additional endpoints addressing patient satisfaction, ease of application/removal, and costs will be included. Pre-formed wound dressings are medical devices. They are approved for marketing with little evidence of their comparative benefits regarding alternatives already existing in the market. Therefore, the choice of the dressing is guided mainly by theoretical properties and personal experiences of its use. Thus, there is a need to generate robust and unbiased evidence of the potential advantages of these new dressings with respect to the conventional ones before extending their use to clinical practice. It is also relevant to evaluate the costs associated with their acquisition and with the outcomes. For the latter part, a cost analysis is also planned.

In our study, we will assess four different dressings. Although no formal comparisons among the new dressings will be carried out, it is relevant to include four of the most used pre-formed dressings in our setting in the same study as it will provide indirect comparisons of the advantages and disadvantages of each of them. We consider this an efficient design. According to the review by Sharma et al. [11], most studies compare only two dressings and most do not compare the new ones with the traditional ones that represent a lower cost for the hospital. Only two studies compared more than two dressings [9, 16].

We also intend to perform a subgroup analysis that could provide information on the differential efficacy of each dressing in different types of surgery or types of patients.

One of the main limitations of the study is the lack of blinding. The implementation of alternative procedures to guarantee the blind assessment (such as a blind evaluator) is feasible and desirable [17]; however, this was considered to be logistically too complicated and unfeasible. Although the assessments are performed by nurses and thus could be subject to subjectivity, the assessment of the presence of blister, erythema, and other endpoints will be assessed as a dichotomous variable, being less prone to bias. Assessments may also be biased by inter-observer variability. To overcome this problem, only two nurses on the team are responsible for the assessments during the scheduled ambulatory visits. However, when an unscheduled changing of dressing is carried out during hospitalization, assessments are performed by a variety of nurses, and although they are all trained in the study procedure, the inter-observer variability cannot be ruled out.

We chose, as the main endpoint, a composite variable, including any aspect of the skin that compromises skin integrity. Although this is an artificially built variable, we think it may provide good information on skin integrity. All components of the variable will also be considered as secondary variables and thus no information will be lost. Although the presence of blistering is the most frequently used primary endpoint in clinical trials comparing wound dressings, some authors have also used the concept of wound complication, including blisters, inflammation, leakage, and maceration, in a composite endpoint [13, 16].

We also included outcomes related to comfort, preferences and ease of manipulation by the nurse, time spent on the cure, and number of cures needed. These variables have not been considered in most studies and thus we consider that they add value to this investigation. Studies including these types of measures have shown the impact of the different dressings on the well-being of patients and on overall costs [4, 18, 19]. Surgical procedures may also have an impact on the outcomes measured in this trial. Factors related to surgery that influence wound healing, such as duration of surgery, total time of ischemia, and use of a layer wound closure technique, will be recorded.

The fact that this is a single-center study may be a drawback for the external validity of the results. Our hospital has very low rates of infection reported in recent years and thus it is expected that few infections will be registered in this study. The incidence of other complications such as hemorrhage is not routinely monitored but could be different from other hospitals because of the techniques used in our setting (i.e. the lack of use of Redon). Also, it has to be taken into account that we will only include patients undergoing fast track, with early mobilization and without major complications before surgery, and this is a population in which low rates of complications are expected.

Our main question is whether any of the modern dressings, all of which claim different advantages, is better than the conventional one (traditional occlusive dressing of sterile gauze and non-woven hypoallergenic adhesive tape) that is used as a standard in this type of surgery in our institution and that is cheaper than any of the pre-formed ones. We hypothesize that any or at least some of them are better and that these advantages would justify its use even if the direct costs are higher. As no differences in terms of skin integrity are expected among the new dressings, only exploratory comparisons for the primary endpoint will be performed among them. We foresee that differences between new dressings in secondary endpoints (ease of application and comfort) may be found and also differences depending on the type of surgery (hip or knee) and characteristics of patients (BMI and presence of diabetes). The large sample size will allow us to explore all of these characteristics, and all of these analyses will generate hypotheses. Thus, we consider that this strategy for the analysis will offer more advantages than other strategies based on post-hoc analysis to check where the differences are.

Finally, patients included in this study constitute a large cohort of patients undergoing arthroplasty procedures. Data on the clinical course of these patients and their complications will also be analyzed to explore factors that may be related to better clinical results.

The results of this study will be useful for decision making and not only will give us information on whether the dressings studied contribute to a better outcome of the wound compared with traditional dressings but also may allow us to decide which dressing will offer better results depending on the characteristics of patients.

## Trial status

The trial is ongoing. As of this writing, 170 patients have been included.

## Additional file

Additional file 1: SPIRIT 2013 Checklist: Recommended items to address in a clinical trial protocol and related documents*. (DOC 121 kb)

## Abbreviations

BMI: Body mass index
CRF: Case report form
SPIRIT: Standard Protocol Items: Recommendations for Interventional Trials
THA: Total hip arthroplasty
TKA: Total knee arthroplasty

## References

[1] Glennie RA, Dea N, Street JT (2015). Dressings and drains in posterior spine surgery and their effect on would complications. J Clin Neurosci..

[2] Vince KG (2012). Wound closure. Healing the collateral damage. J Bone Joint Surg Br..

[3] Abboud EC, Settle JC, Legare TB, Marcet JE, Barillo DJ, Sanchez JE (2014). Silver-based dressings for the reduction of surgical site infection: review of current experience and recommendation for future studies. Burns.

[4] Abejon-Arroyo A, López-Casanova P, Verdú-Soriano J, Torra I, Bou JE (2015). Open-label clinical trial comparing the clinical and economic effectiveness of using a polyurethane film surgical dressing with gauze surgical dressings in the care of post-operative surgical wounds. Int Wound J..

[5] Jester R, Russell R, Fell J, Williams S, Prest C (2000). A one hospital study of the effect of wound dressings and other related factors on skin blistering following total hip and knee arthroplasty. J Orthop Nursing.

[6] Aindow D, Butcher M (2005). Films or fabrics: is it time to re-appraise postoperative dressings?. Br J Nurs..

[7] Bredow J, Oppermann J, Hoffmann K, Hellmich M, Wenk B, Simons M (2015). Clinical trial to evaluate the performance of a flexible self-adherent absorbent dressing coated with a soft silicone layer compared to a standard wound dressing after orthopedic or spinal surgery: study protocol for a randomized controlled trial. Trials.

[8] Halawi MJ (2015). Fracture blisters after primary total knee arthroplasty. Am J Orthop. (Belle Mead, NJ)..

[9] Cosker T, Elsayed S, Gupta S, Mendonca AD, Tayton KJJ (2005). Choice of dressing has a major impact on blistering and healing outcomes in orthopaedic patients. J Wound Care.

[10] Dillon JM, Clarke JV, Deakin AH, Nico AC, Kinninmonth AWG (2007). Correlation of total knee replacement surgery wound dynamic morphology and dressing material properties. J Biomech..

[11] Sharma G, Lee SW, Atanacio O, Parvizi J, Kim TK (2017). In search of the optimal wound dressing material following total hip and knee arthroplasty: a systematic review and meta-analysis. Int Orthop..

[12] Ousey K, Gillibrand W, Stephenson J (2013). Achieving international consensus for the prevention of orthopaedic wound blistering: results of a Delphi survey. Int Wound J..

[13] Springer BD, Beaber WB, Griffin WL, Mason JB, Odum SM (2015). Role of surgical dressings in total joint arthroplasty: a randomized controlled trial. Am J Orthop. (Belle Mead, NJ)..

[14] Clarke JV, Deakin AH, Dillon JM, Emmerson S, Kinninmonth AW (2009). A prospective clinical audit of a new dressing design for lower limb arthroplasty wounds. J Wound Care.

[15] Chan AW, Tetzlaff JM, Altman DG, Laupacis A, Gøtzsche PC, Krleža-Jerić K (2013). SPIRIT 2013 statement: defining standard protocol items for clinical trials. Ann Intern Med..

[16] Dobbelaere A, Schuermans N, Smet S, Van Der Straeten C, Victor J (2015). Comparative study of innovative postoperative wound dressings after total knee arthroplasty. Acta Orthop Belg..

[17] Koval KJ, Egol KA, Hiebert R, Spratt KF (2007). Tape blisters after hip surgery: can they be eliminated completely?. Am J Orthop. (Belle Mead, NJ).

[18] Abuzakuk TM, Coward P, Shenava Y, Kumar VS, Skinner JA (2006). The management of wounds following primary lower limb arthroplasty: a prospective, randomised study comparing hydrofibre and central pad dressings. Int Wound J..

[19] Cai J, Karam JA, Parvizi J, Smith, Sharkey PB (2014). Aquacel surgical dressing reduces the rate of acute PJI following total joint arthroplasty: a case-control study. J Arthroplast..

